# Supplementary figures and images for: Intensity Differences of Resistance Training for Type 2 Diabetic Patients: A Systematic Review and Meta-Analysis
Source: Healthcare (Basel). 2023 Feb 3;11(3):440. doi: 10.3390/healthcare11030440 (PMC9914423; doi:10.3390/healthcare11030440)

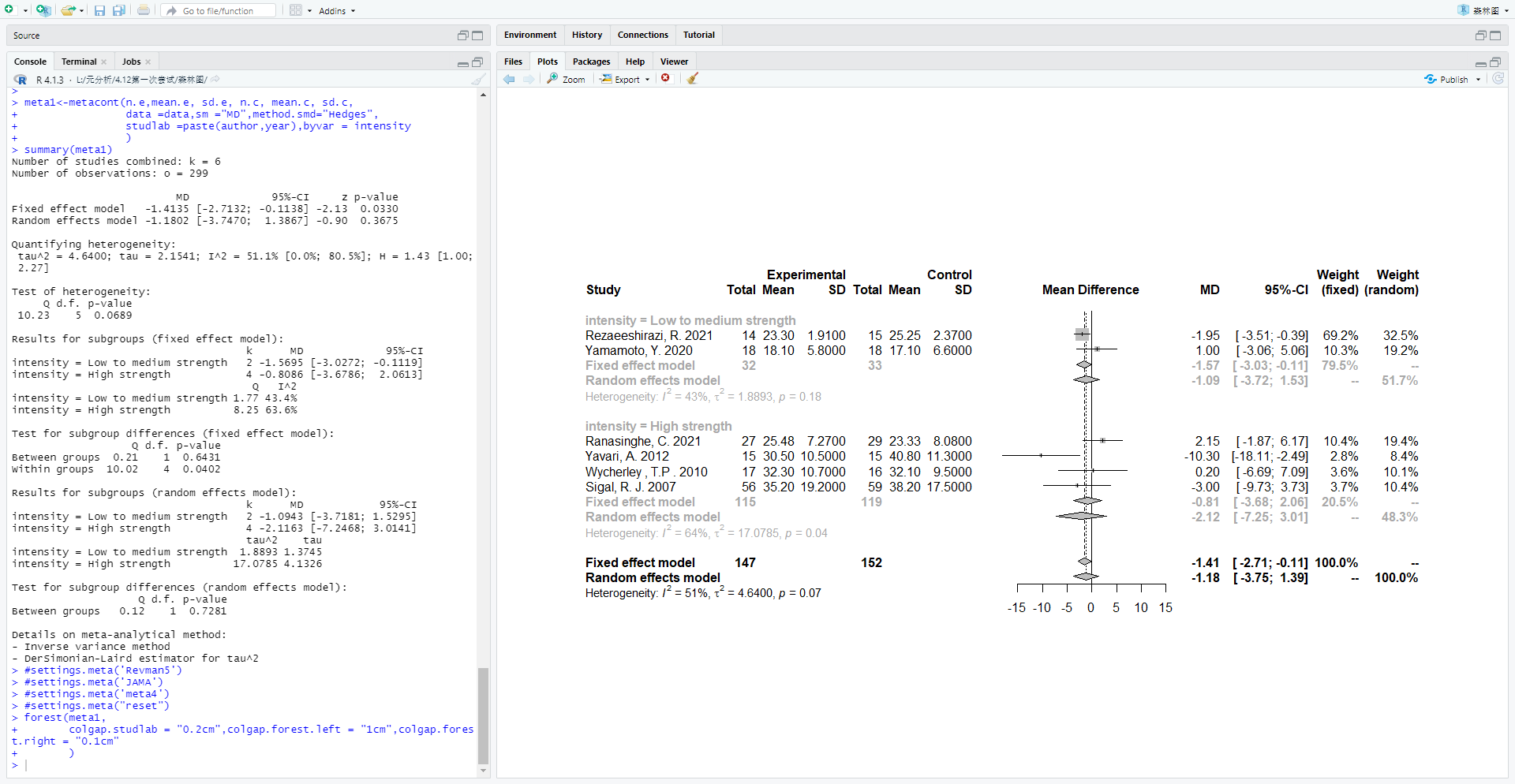

Supplement: Supplementary file 1 [file healthcare-11-00440-s001.zip › Screenshot of subgroup analysis for Meta-analysis (source of data in Table 2)/BF.png]

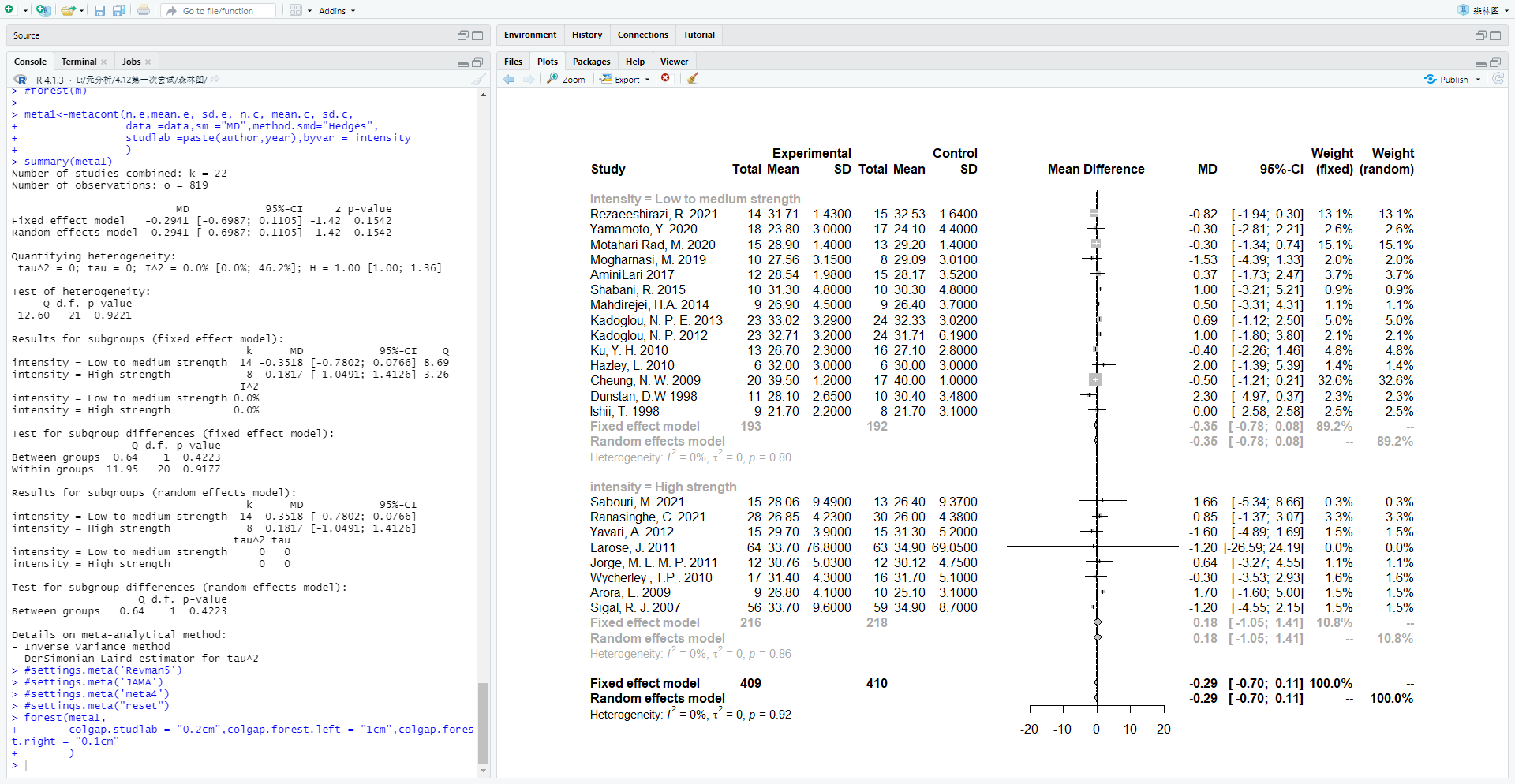

Supplement: Supplementary file 1 [file healthcare-11-00440-s001.zip › Screenshot of subgroup analysis for Meta-analysis (source of data in Table 2)/BMI.png]

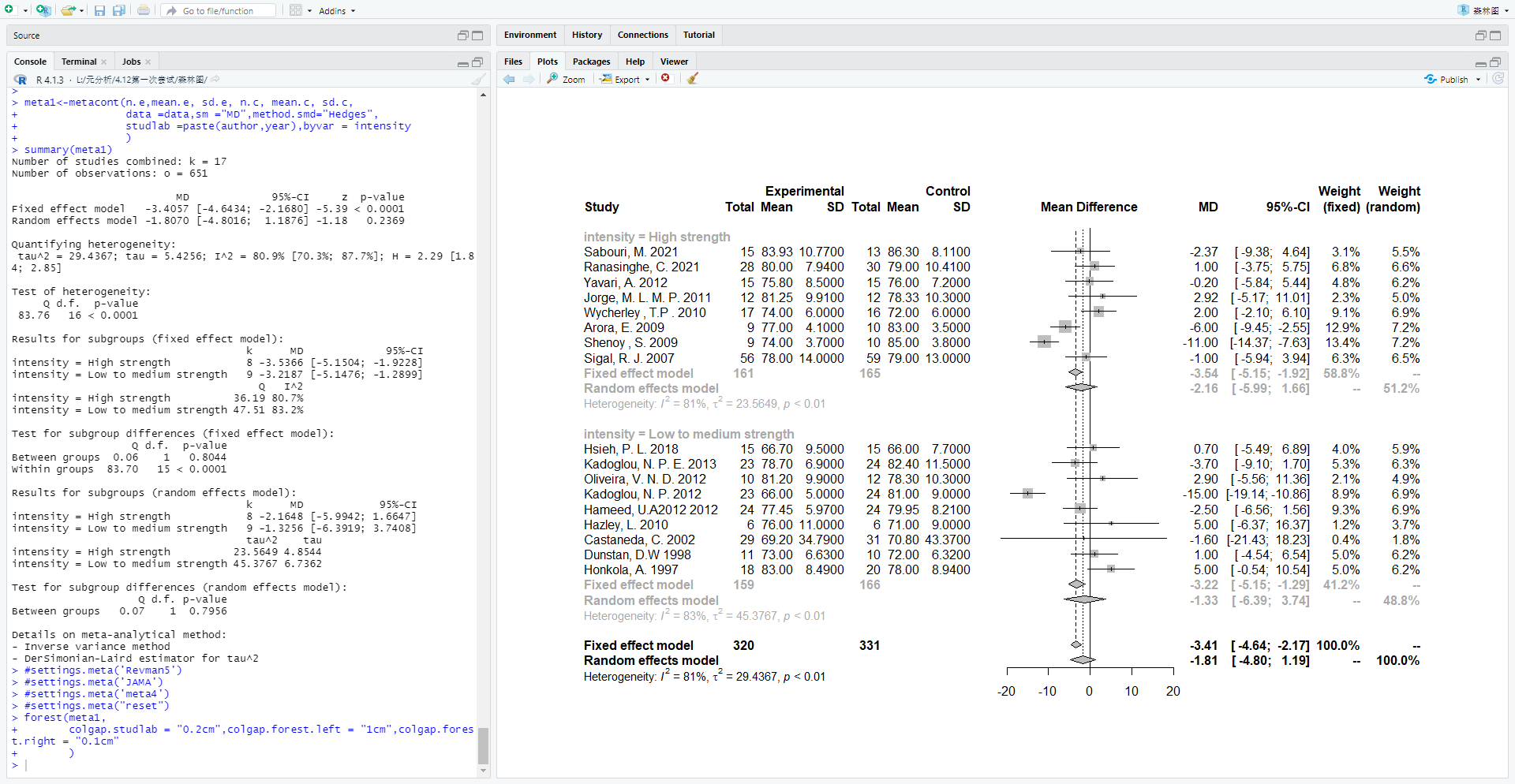

Supplement: Supplementary file 1 [file healthcare-11-00440-s001.zip › Screenshot of subgroup analysis for Meta-analysis (source of data in Table 2)/DBP.png]

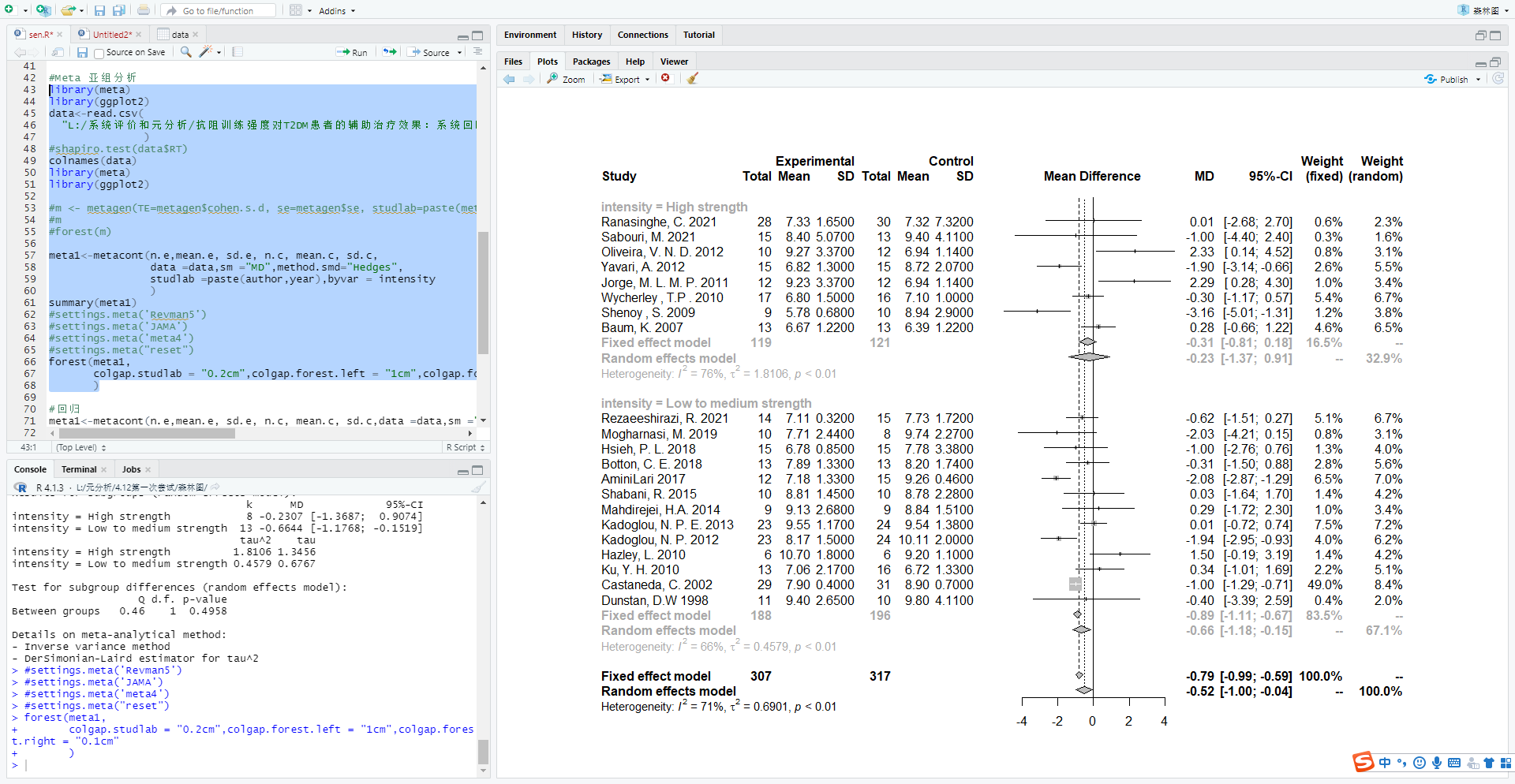

Supplement: Supplementary file 1 [file healthcare-11-00440-s001.zip › Screenshot of subgroup analysis for Meta-analysis (source of data in Table 2)/FBG.png]

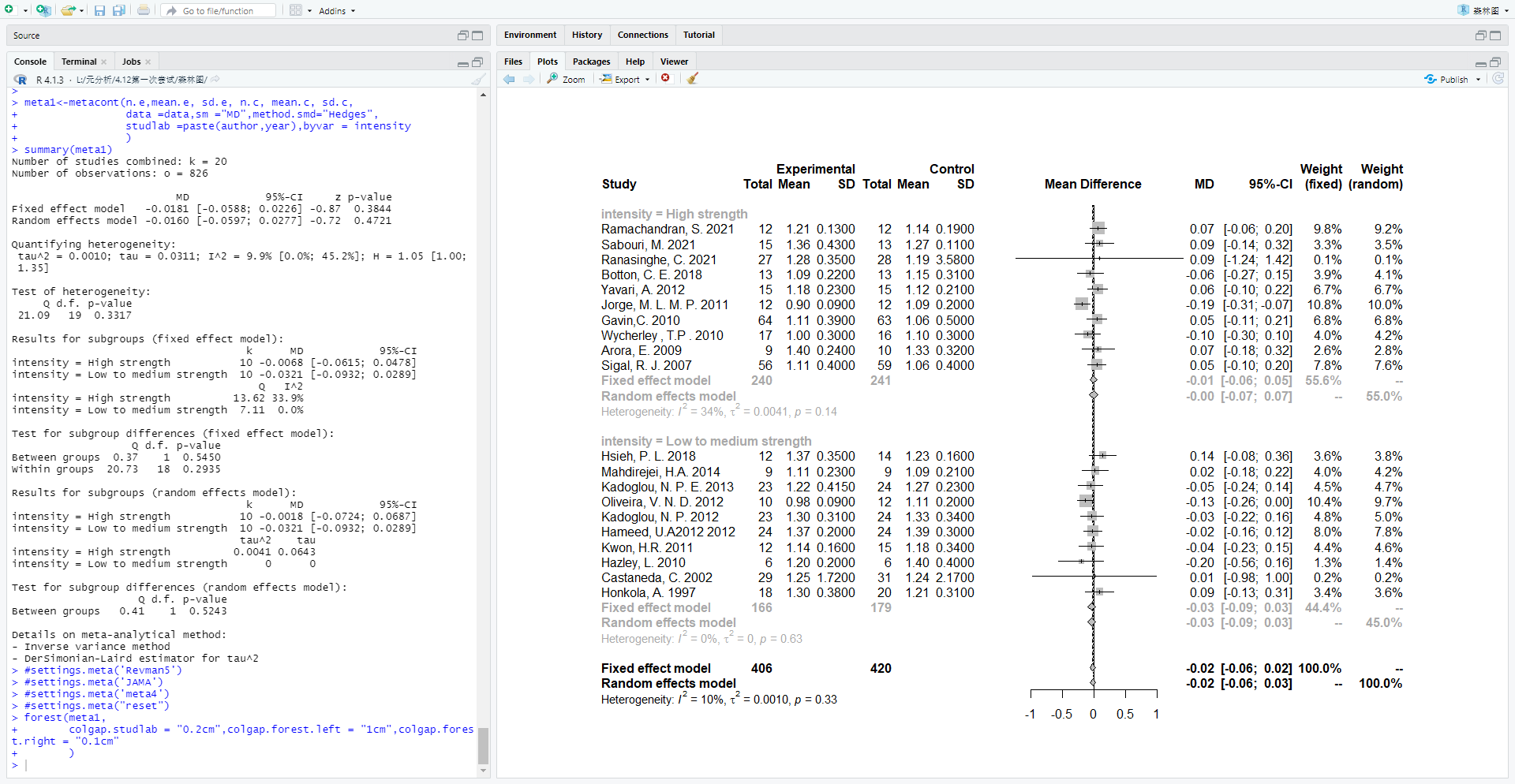

Supplement: Supplementary file 1 [file healthcare-11-00440-s001.zip › Screenshot of subgroup analysis for Meta-analysis (source of data in Table 2)/HDL.png]

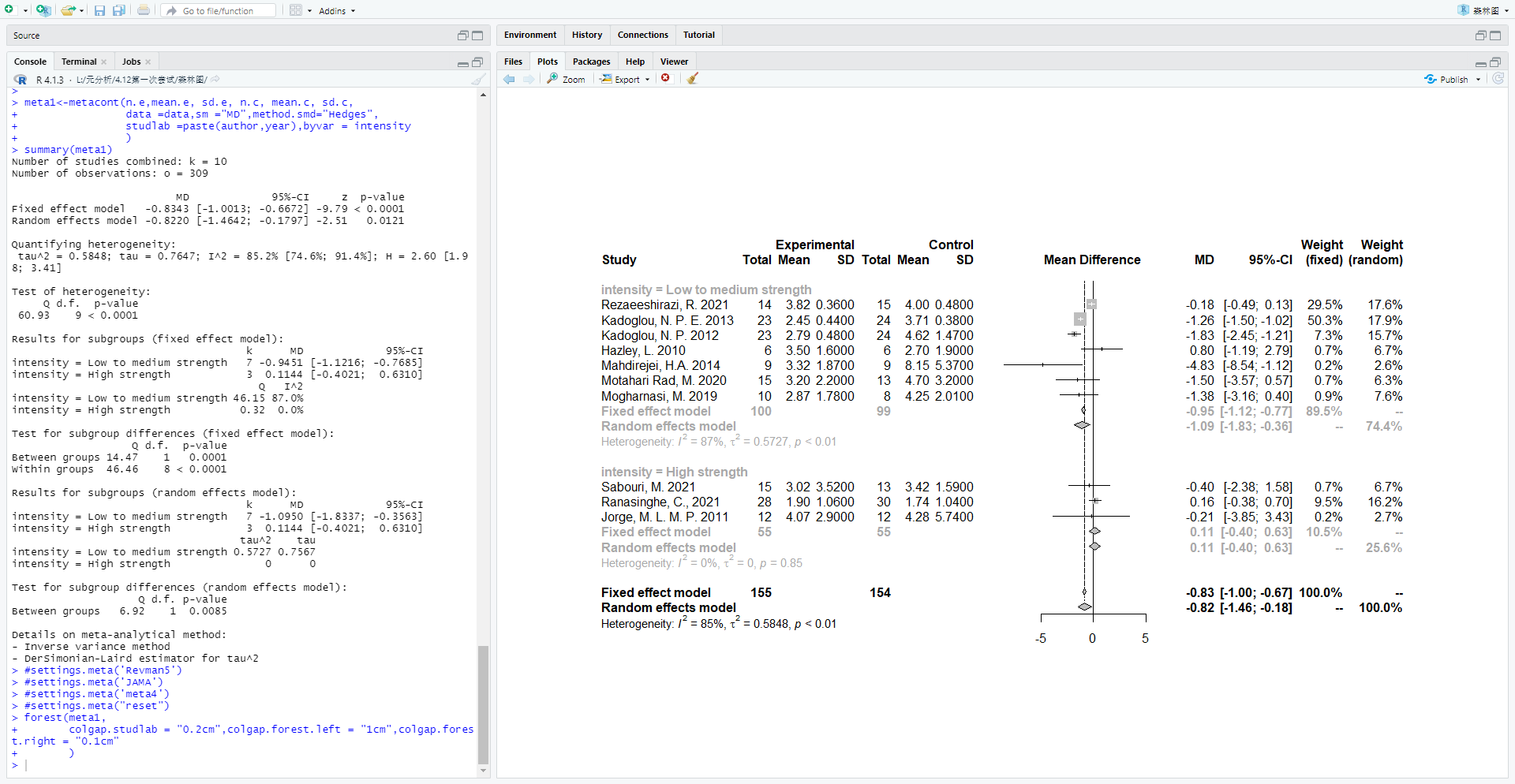

Supplement: Supplementary file 1 [file healthcare-11-00440-s001.zip › Screenshot of subgroup analysis for Meta-analysis (source of data in Table 2)/HOMA.png]

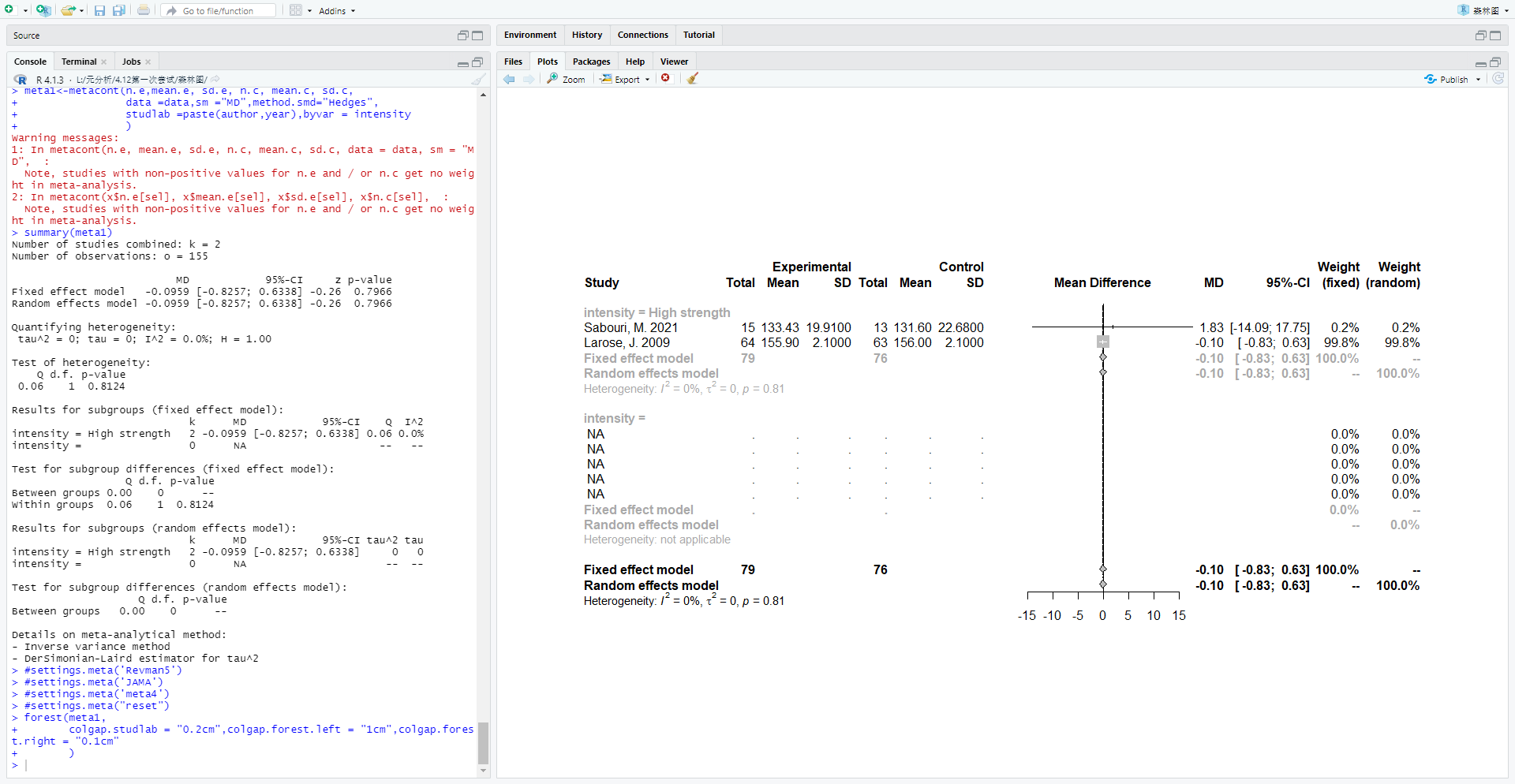

Supplement: Supplementary file 1 [file healthcare-11-00440-s001.zip › Screenshot of subgroup analysis for Meta-analysis (source of data in Table 2)/HRmax.png]

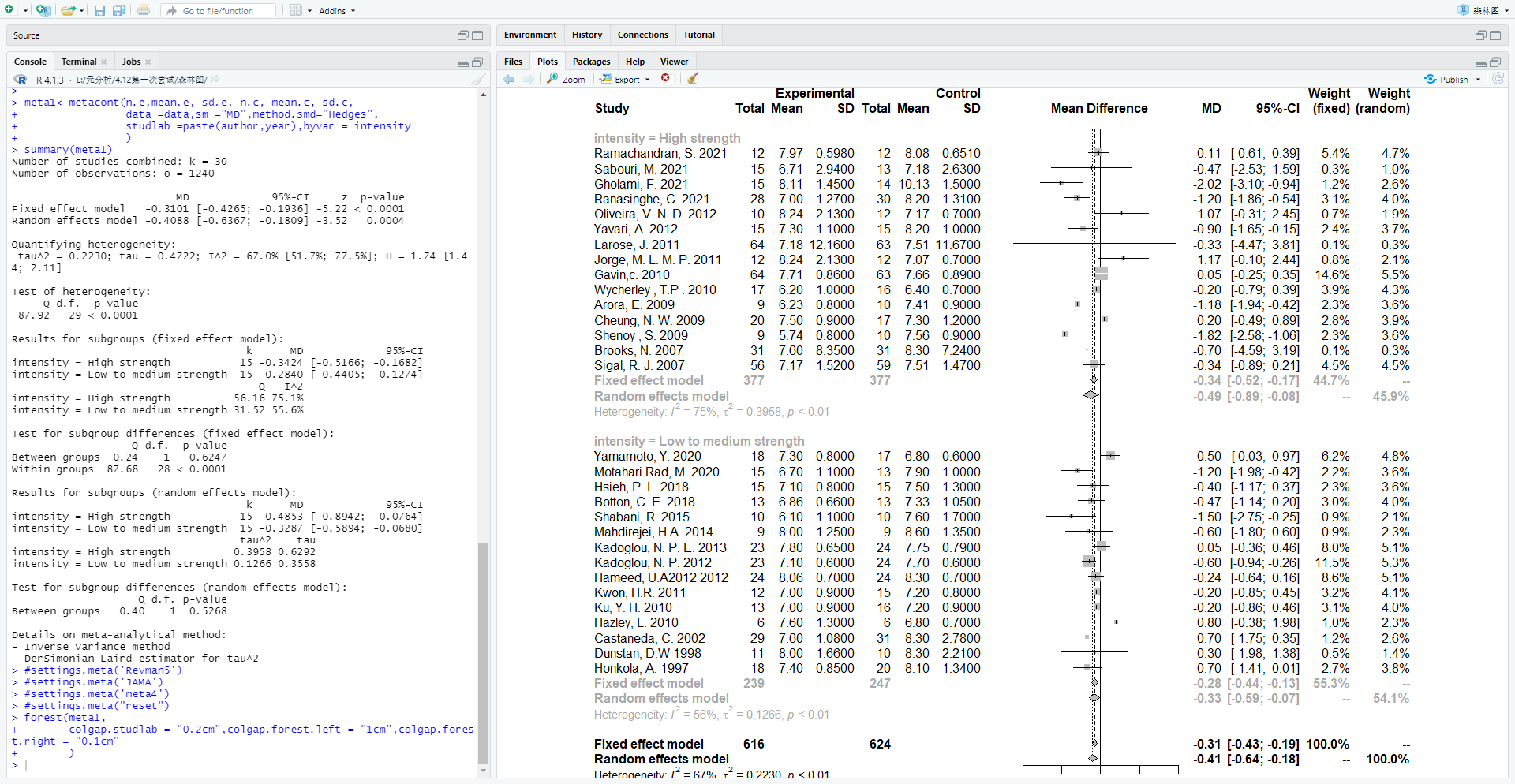

Supplement: Supplementary file 1 [file healthcare-11-00440-s001.zip › Screenshot of subgroup analysis for Meta-analysis (source of data in Table 2)/Hba1c.png]

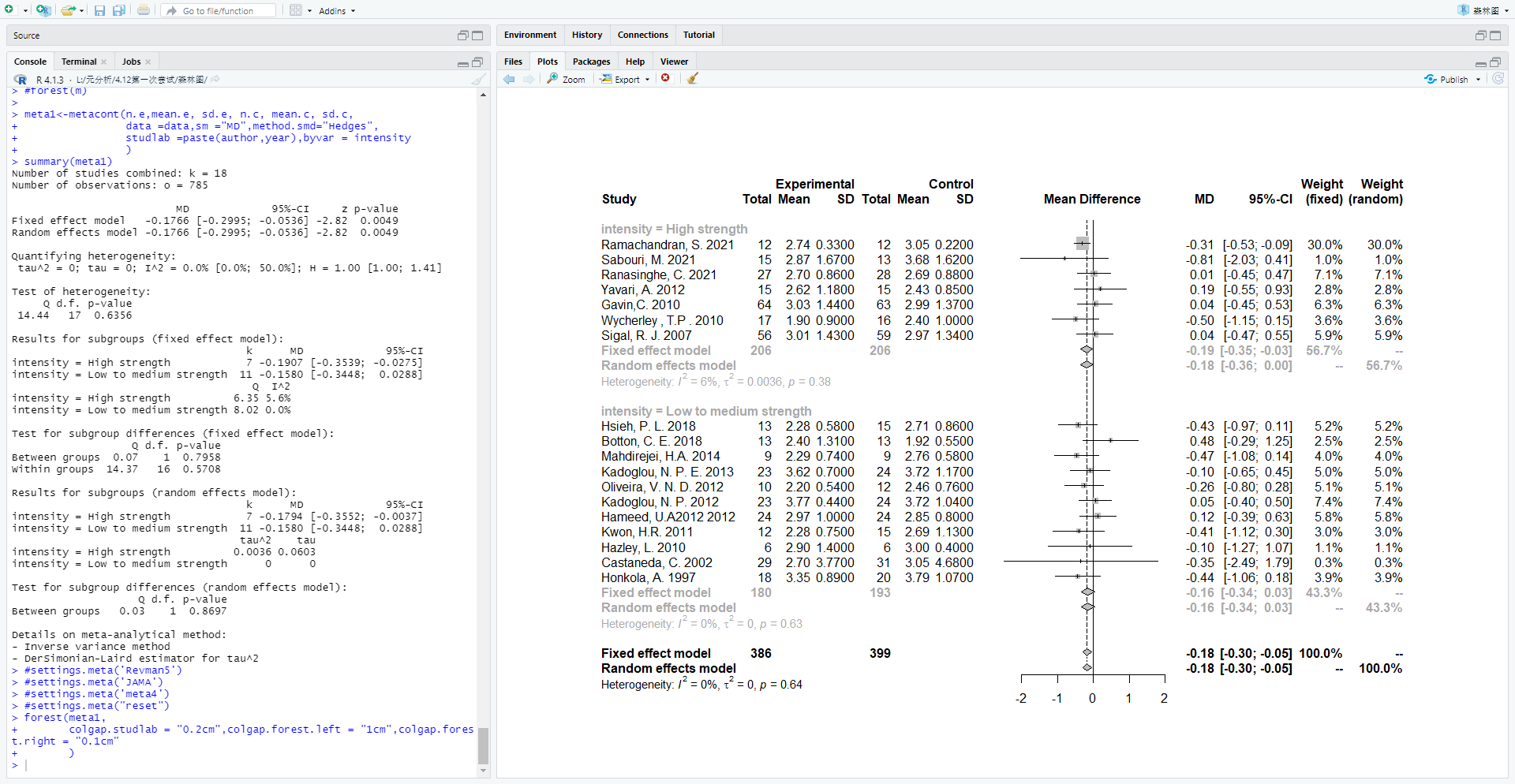

Supplement: Supplementary file 1 [file healthcare-11-00440-s001.zip › Screenshot of subgroup analysis for Meta-analysis (source of data in Table 2)/LDL.png]

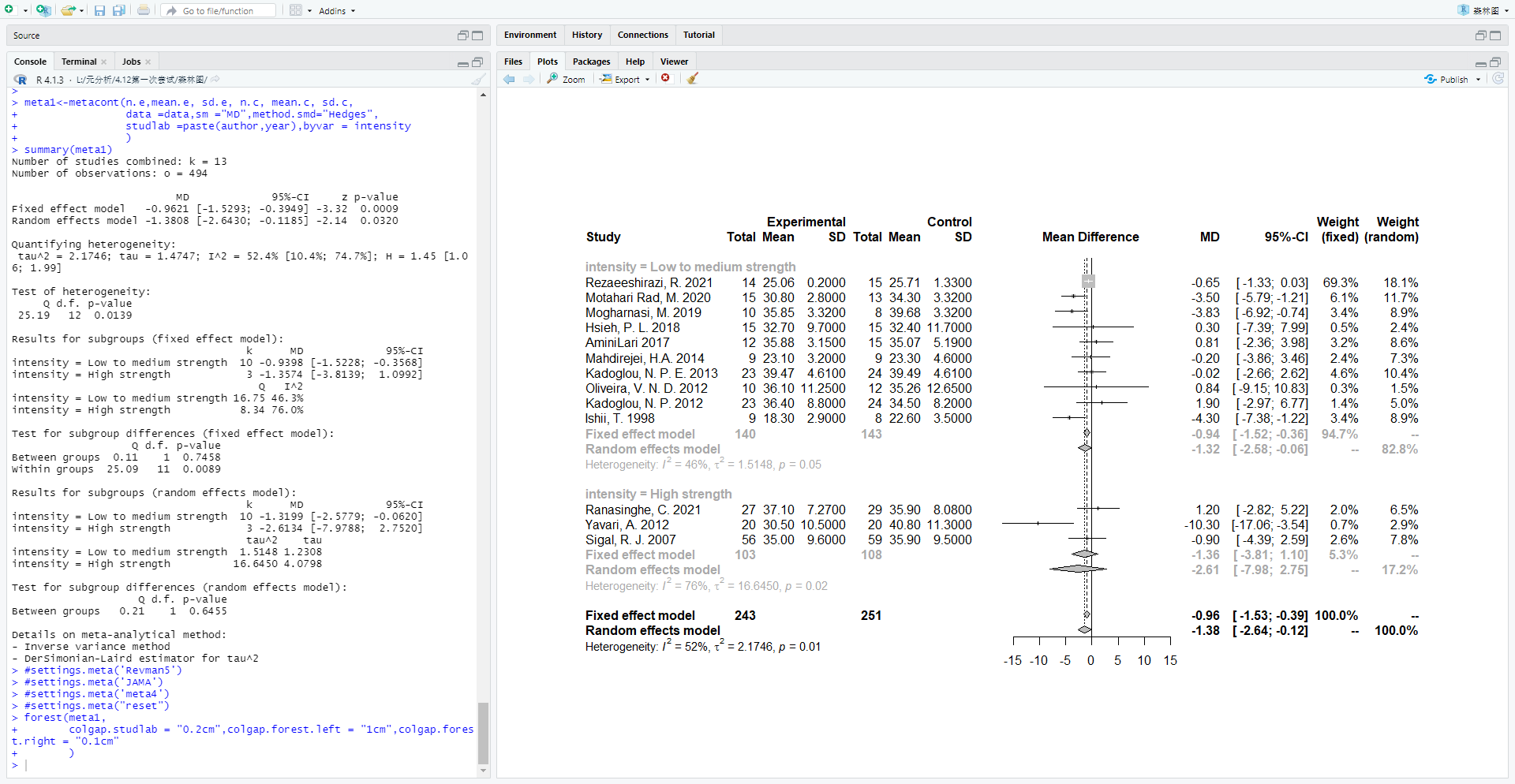

Supplement: Supplementary file 1 [file healthcare-11-00440-s001.zip › Screenshot of subgroup analysis for Meta-analysis (source of data in Table 2)/PBF.png]

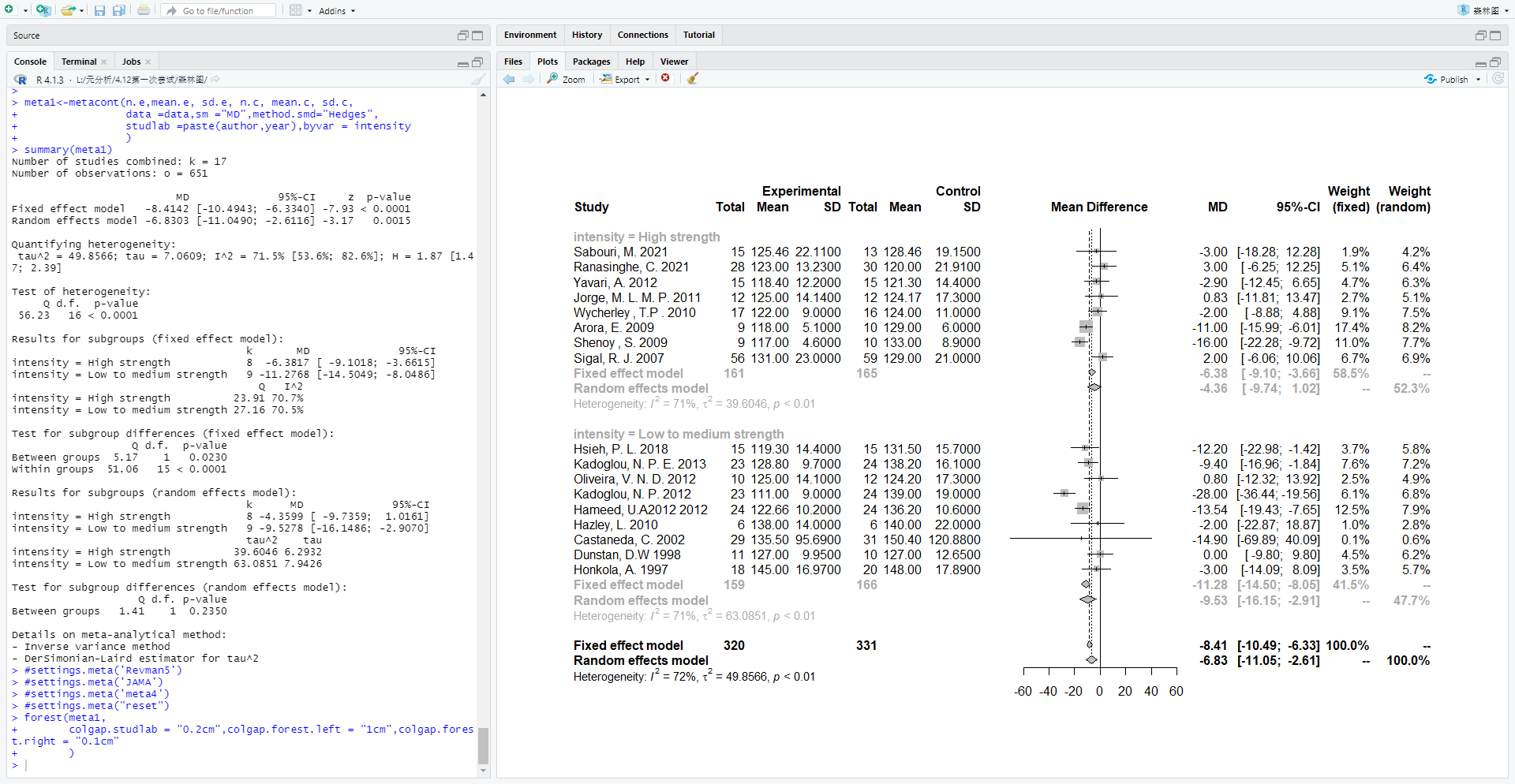

Supplement: Supplementary file 1 [file healthcare-11-00440-s001.zip › Screenshot of subgroup analysis for Meta-analysis (source of data in Table 2)/SBP.png]

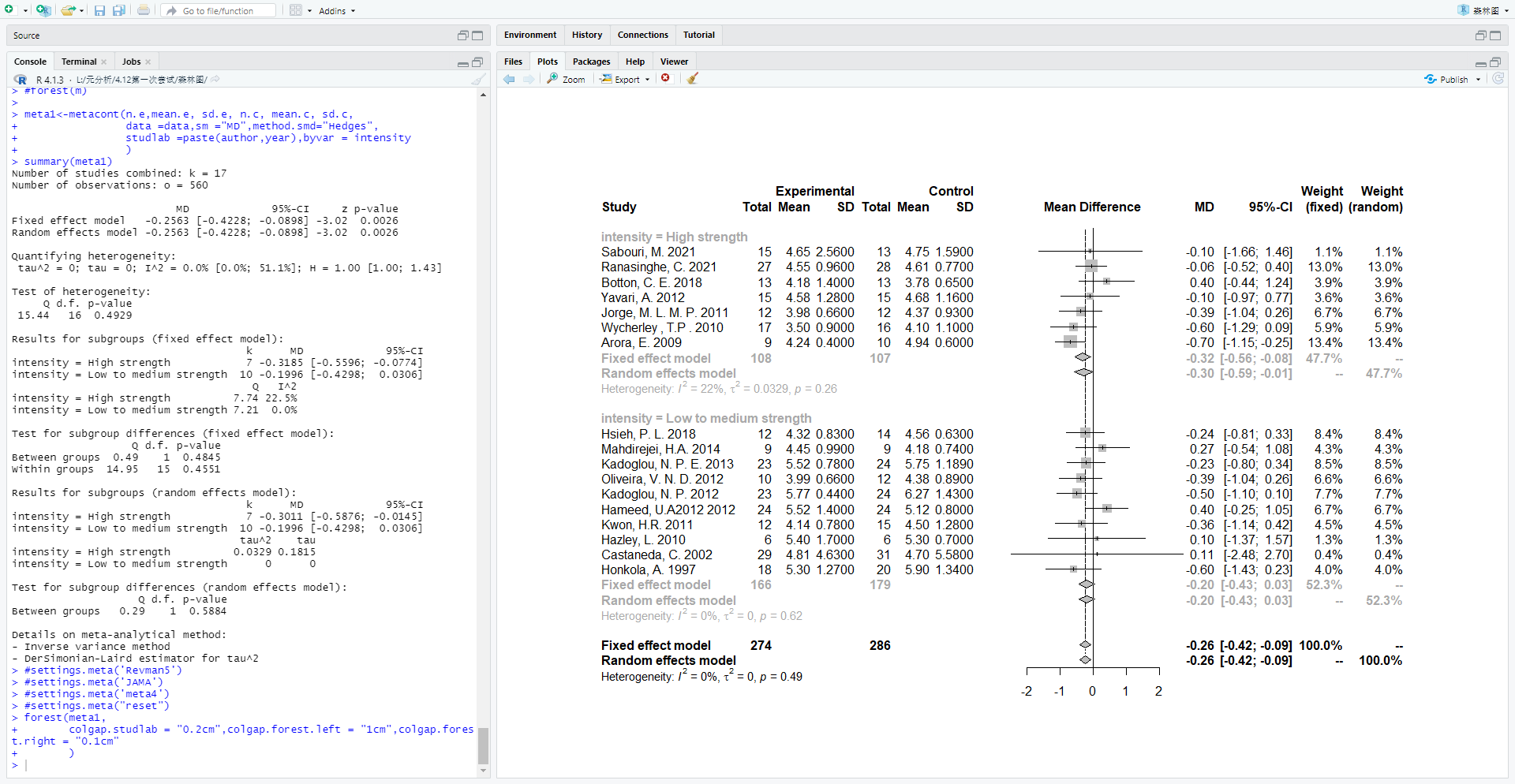

Supplement: Supplementary file 1 [file healthcare-11-00440-s001.zip › Screenshot of subgroup analysis for Meta-analysis (source of data in Table 2)/TC.png]

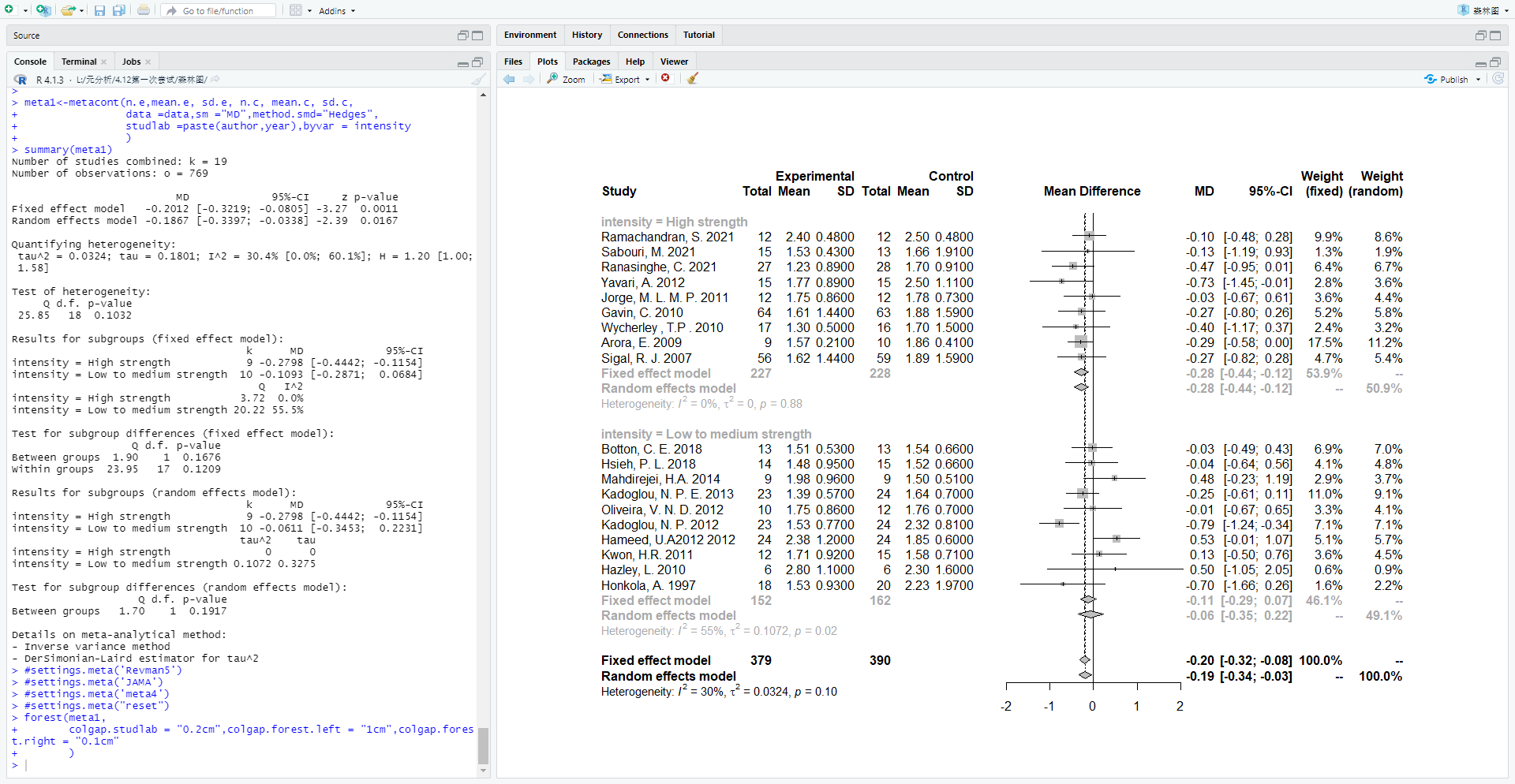

Supplement: Supplementary file 1 [file healthcare-11-00440-s001.zip › Screenshot of subgroup analysis for Meta-analysis (source of data in Table 2)/TG.png]

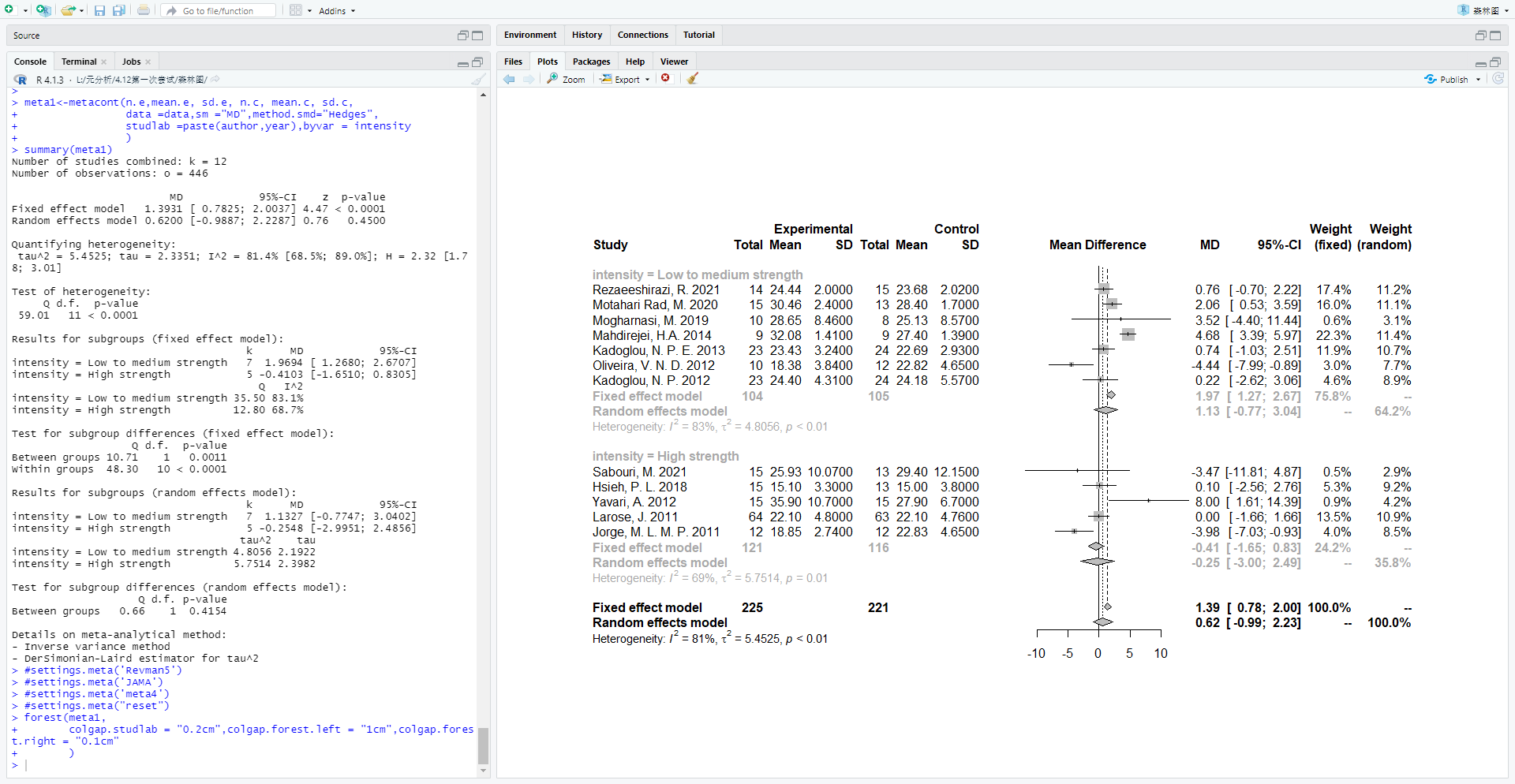

Supplement: Supplementary file 1 [file healthcare-11-00440-s001.zip › Screenshot of subgroup analysis for Meta-analysis (source of data in Table 2)/VO2max.png]

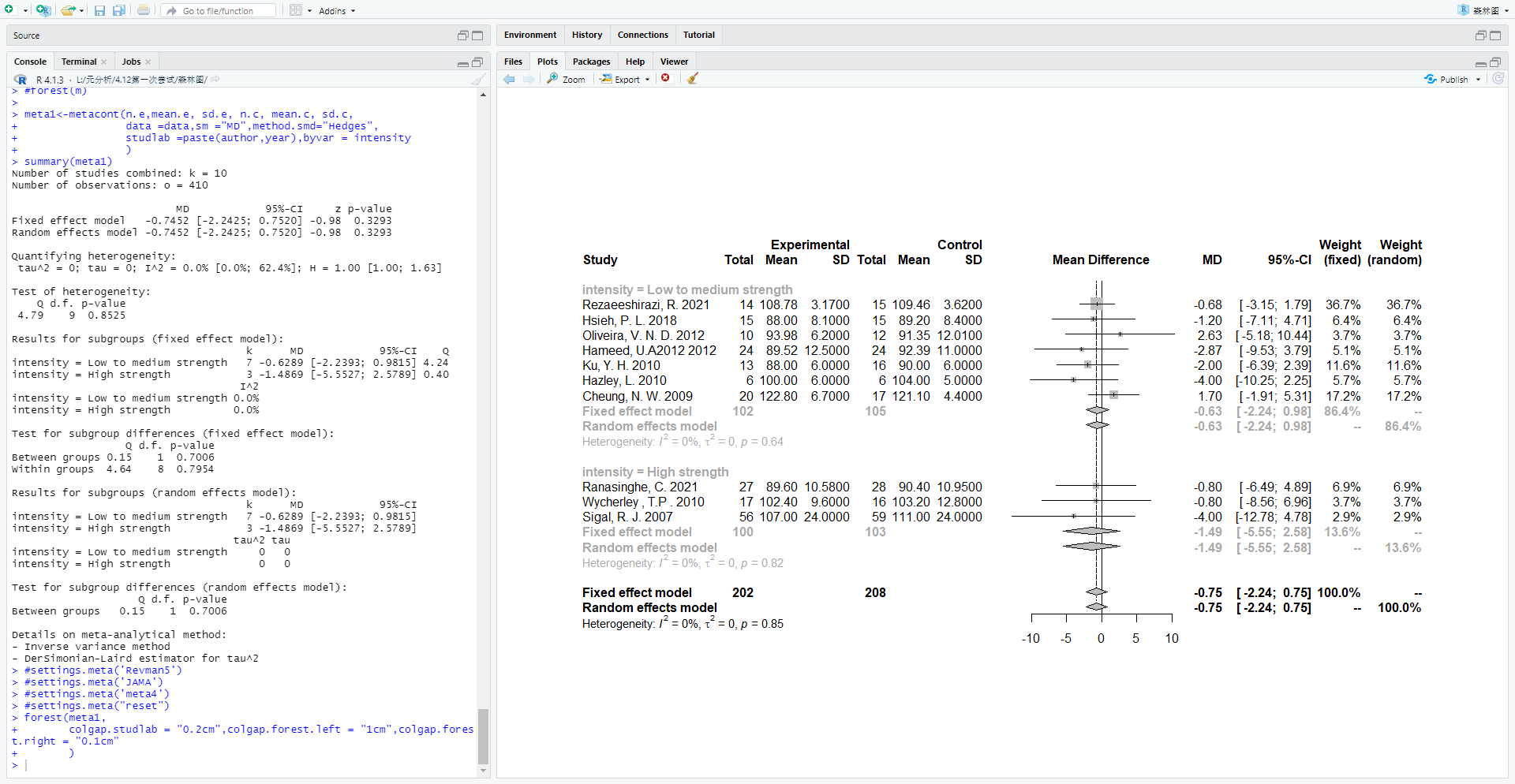

Supplement: Supplementary file 1 [file healthcare-11-00440-s001.zip › Screenshot of subgroup analysis for Meta-analysis (source of data in Table 2)/WC.png]

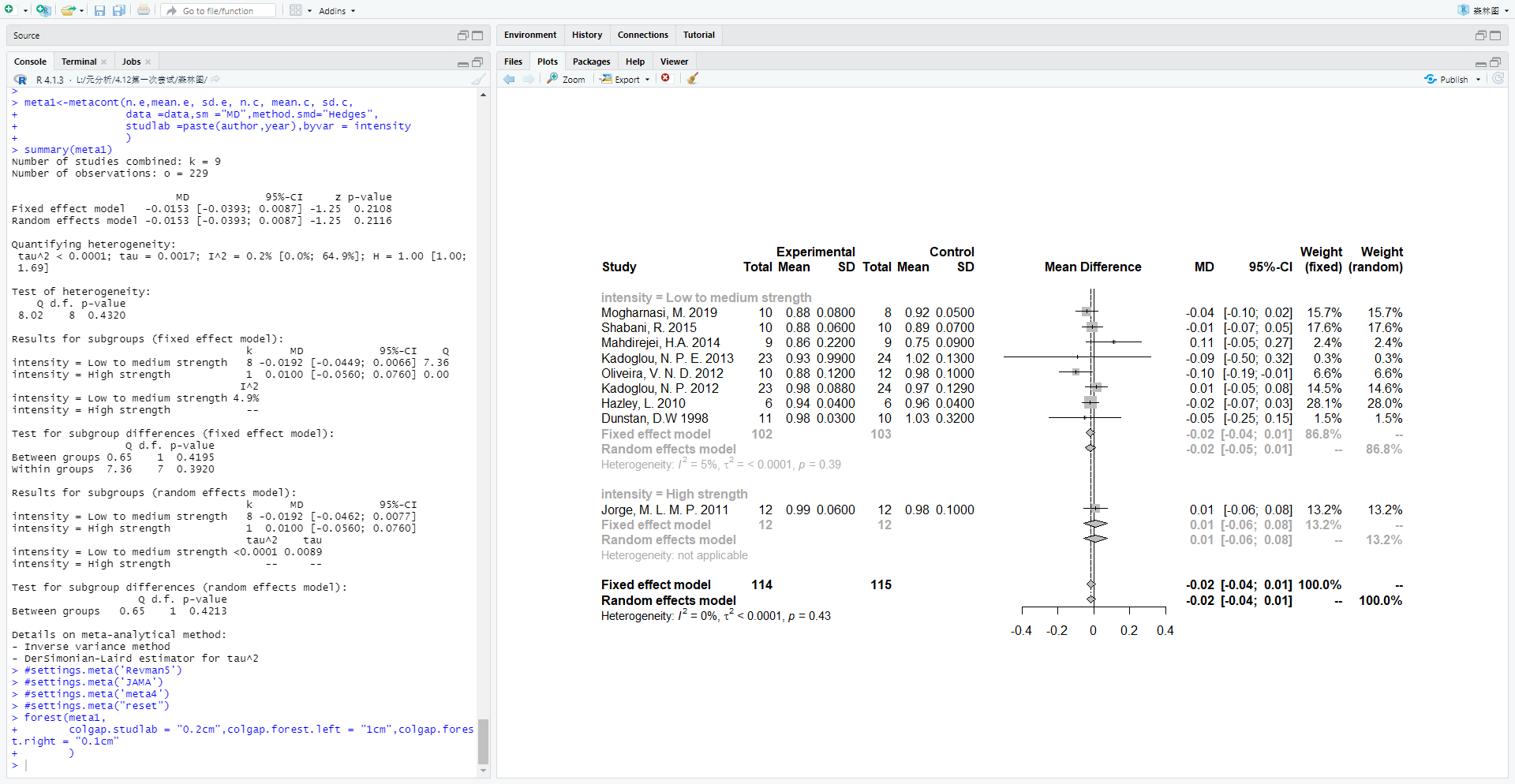

Supplement: Supplementary file 1 [file healthcare-11-00440-s001.zip › Screenshot of subgroup analysis for Meta-analysis (source of data in Table 2)/WHR.png]

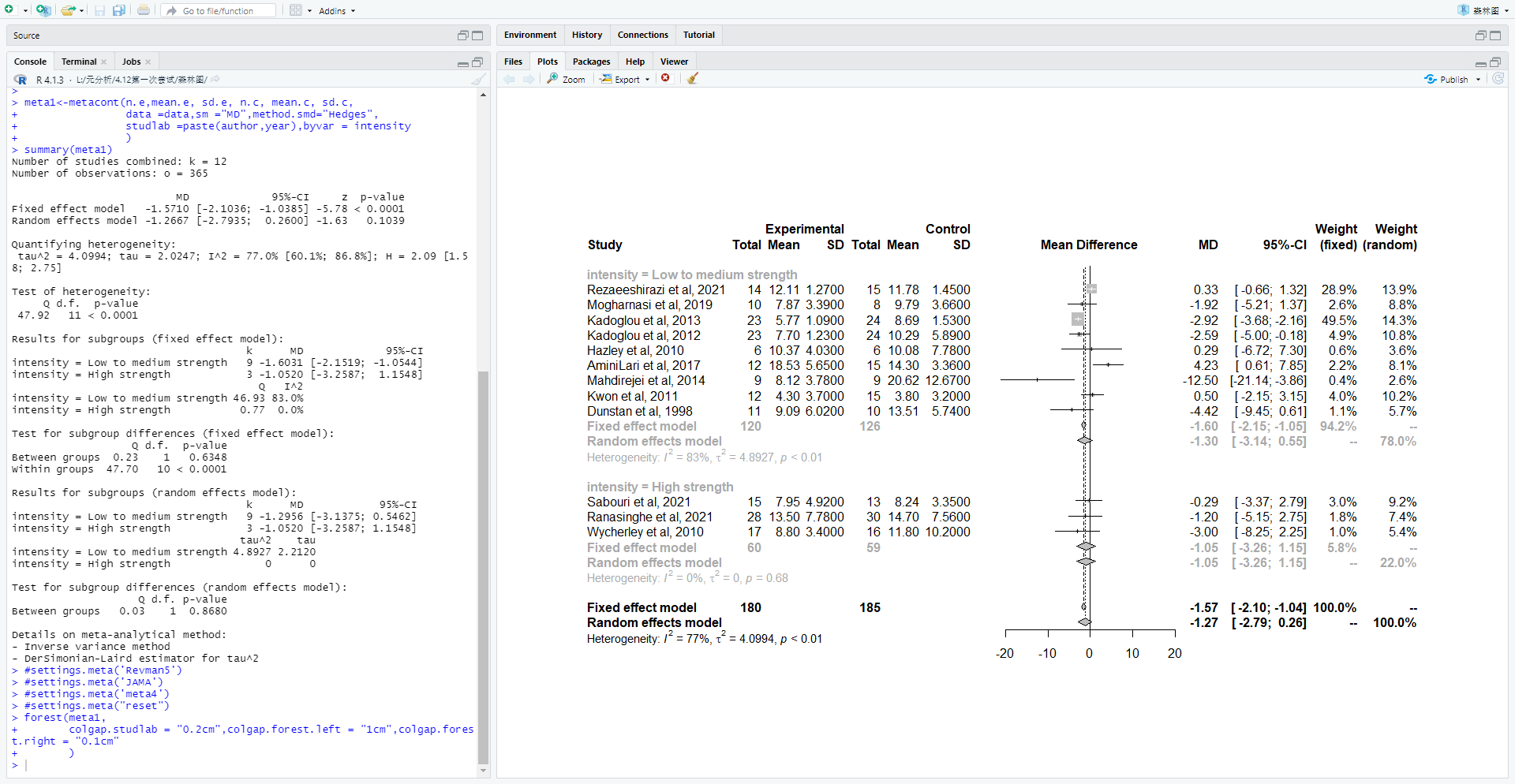

Supplement: Supplementary file 1 [file healthcare-11-00440-s001.zip › Screenshot of subgroup analysis for Meta-analysis (source of data in Table 2)/insulin.png]

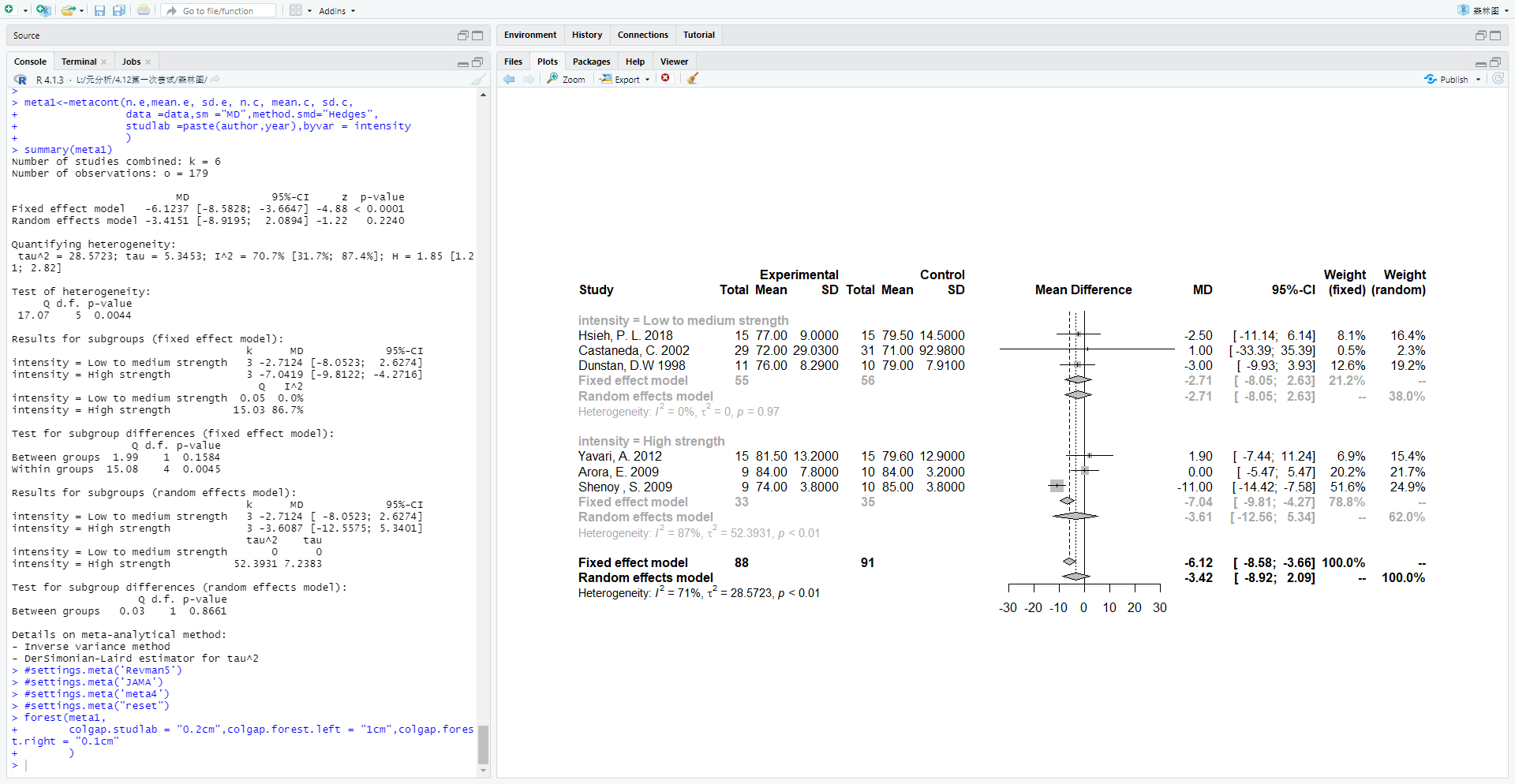

Supplement: Supplementary file 1 [file healthcare-11-00440-s001.zip › Screenshot of subgroup analysis for Meta-analysis (source of data in Table 2)/restHR.png]

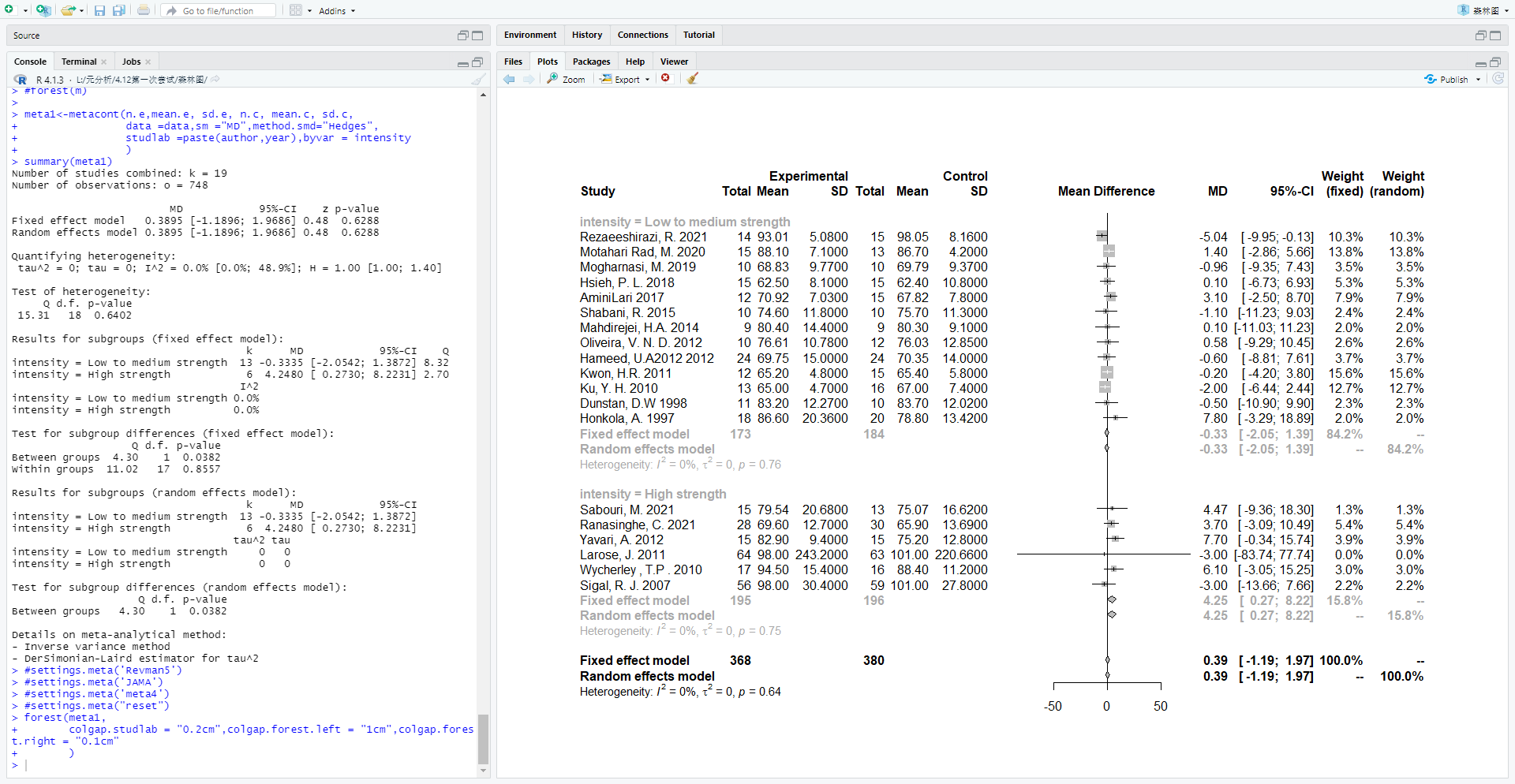

Supplement: Supplementary file 1 [file healthcare-11-00440-s001.zip › Screenshot of subgroup analysis for Meta-analysis (source of data in Table 2)/weight.png]
